# Prognostic impact of the Controlling Nutritional Status Score in patients with biliary tract cancer: a systematic review and meta-analysis

**DOI:** 10.3389/fonc.2023.1240008

**Published:** 2023-08-17

**Authors:** Zhuoran Liu, Haoge Zhou, Yu Zhou, Menglin Yu, Yonglang Cheng, Jing Li

**Affiliations:** ^1^ Department of General Surgery (Hepatopancreatobiliary Surgery), The Affiliated Hospital of Southwest Medical University, Luzhou, Sichuan, China; ^2^ Department of Vascular Surgery, The Affiliated Hospital of Southwest Medical University, Luzhou, Sichuan, China

**Keywords:** meta-analysis, biliary tract cancer, prognosis, controlling nutritional status score, clinical use

## Abstract

**Background:**

Biliary tract cancer (BTC) is a malignancy associated with unfavorable outcomes. Advanced BTC patients have a propensity to experience compromised immune and nutritional status as a result of obstructive jaundice and biliary inflammation. Currently, there is a lack of consensus on the impact of the Controlling Nutritional Status (CONUT) score in the context of BTC prognosis. The purpose of this study is to conduct a meta-analysis on the association between CONUT and the prognosis of patients suffering from BTC.

**Methods:**

A defined search strategy was implemented to search the PubMed, Embase, and Web of Science databases for eligible studies published until March 2023, with a focus on overall survival (OS), relapse-free survival/recurrence-free survival(RFS), and relevant clinical characteristics. The prognostic potential of the CONUT score was evaluated using hazard ratios (HRs) or odds ratios (ORs) with 95% confidence intervals (CIs).

**Results:**

In this meta-analysis, a total of 1409 patients from China and Japan were involved in 9 studies. The results indicated that the CONUT score was significantly correlated with worse OS (HR=2.13, 95% CI 1.61-2.82, *P*<0.0001) and RFS (HR=1.83, 95% CI 1.44–2.31, *P*<0.0001) in patients with BTC. And, the analysis showed that a high CONUT score was significantly associated with clinical characteristics such as jaundice (OR=1.60, 95% CI=1.14–2.25, *P*=0.006), poorly differentiated tumor (OR=1.43, 95% CI=1.03–1.99, *P*=0.03), pT3 and 4 stage of the tumor (OR=1.87, 95% CI=1.30–2.68, *P*=0.0007), and complications of Clavien-Dindo classification grade IIIa or higher (OR=1.79, 95% CI=1.03–3.12, *P*=0.04).

**Conclusion:**

This meta-analysis indicates that a high CONUT score can serve as a significant prognostic indicator for survival outcomes among patients diagnosed with BTC.

## 1 Introduction

Biliary tract cancer (BTC) including cholangiocarcinoma, gallbladder cancer, and ampulla of Vater cancer (1), represents a significant challenge in clinical practice. BTC is a rare global occurrence, exhibiting an extremely unfavorable prognosis, with a substantially higher incidence observed in low-income countries compared to their high-income counterparts (2, 3). Currently, there is a widespread consensus that surgical intervention is the primary therapeutic modality for patients diagnosed with BTC. The prognosis for BTC patients, however, is notably unfavorable and the 5-year overall survival (OS) rate is estimated to be less than 20% when all stages of the disease are considered. Patients with advanced BTC often exhibit declining immune-nutritional status due to pathophysiological changes induced by obstructive jaundice and inflammation of the biliary tract (4, 5). Thus, the utilization of immune nutritional markers can facilitate precise risk stratification and forecast the optimal surgical intervention and treatment for BTC patients, proving to be an invaluable approach.

The Controlling Nutritional Status (CONUT) score is a self-sufficient tool for nutritional assessment, which was first established by Ignacio et al. (6). It is computed by analyzing three variables: serum albumin concentration, cholesterol level, and peripheral lymphocyte count. The CONUT score is then categorized into four graded levels based on total points, which include normal (0-1 points), mild (2-4 points), moderate (5-8 points), and severe (9-12 points) (**Table 1**)

**Table 1:** The scoring criteria for the CONUT score.

Parameters	Degree			
	Normal	Light	Moderate	Severe
Total serum cholesterol(mg/dL)	≥180	140-180	100-139	<100
Score	0	1	2	3
Total lymphocyte count(/mm^3^)	≥1600	1200-1599	800-1199	<800
Score	0	1	2	3
Serum albumin(g/dL)	3.50-4.50	3.00-3.49	2.50-2.99	<2.50
Score	0	2	4	6

In recent years, several studies have demonstrated the prognostic significance of the CONUT score in patients with malignant tumors (7–10). More recently, a multitude of investigations have investigated the relationship between the CONUT score and prognosis in individuals with BTC. Many of these studies have found that the CONUT score is an independent prognostic factor in patients with BTC. However, there remains a lack of consensus regarding the prognostic value of the CONUT score in this patient population. So, this meta-analysis’s objective was to comprehensively evaluate the associations between the CONUT score and clinical outcomes in individuals with BTC, drawing from all relevant available research.

## 2 Method

### 2.1 Literature search

The search was conducted according to the Preferred Reporting Items for Systematic Reviews and Meta-Analyses guidelines. Systematic searches were conducted in the PubMed, Embase, and Web of Science databases to identify all relevant studies published before March 2023 to evaluate the prognostic value of the CONUT score in BTC. using the following search items: “Controlling Nutritional Status”, “CONUT”, “CONUT score”, “biliary tract cancer”, “bile duct cancer”, “bile duct neoplasms”, “cholangiocarcinoma”, “gallbladder cancer”, “Vater ampullary carcinoma” and “Ampulla of Vater”. All searches were performed using a combination of MeSH terms and free-text words. The publication language was limited to English. References within the identified articles were manually examined to identify other potentially eligible studies. This meta-analysis has been registered in PROSPERO (http://www.crd.york.ac.uk/PROSPERO) with registration number CRD42023424382.

### 2.2 Inclusion and exclusion criteria

After retrieving relevant articles using specified search terms, articles were screened based on the following selection criteria: (i) articles specifically addressing the predictive significance of the CONUT score in patients with BTC; (ii) patients diagnosed with BTC and divided into two groups; (iii) availability of hazard ratios (HRs) and their corresponding 95% confidence intervals (CIs) and *P*-values for OS, relapse-free survival/recurrence-free survival(RFS) or other relevant effect metrics; (iv) articles published in full text. In addition, retrieved articles meeting any of the following criteria were excluded: previous reviews, letters, case reports, conference abstracts, comments, meta-analyses, books or documents, and unpublished articles. Two authors (ZL and YZ) independently assessed the eligibility of studies based on the aforementioned criteria, with any disagreements resolved through consultation with a third author (HZ).

### 2.3 Quality assessment and data extraction

The selected studies were subject to data extraction of crucial information, such as the first author’s name, publication year, duration, country, sample size, tumor type, study design, treatment method, CONUT score cut-off, study endpoints, and survival data, which includes outcome type, analysis method, HRs, and corresponding 95% CIs. Given the superior accuracy of multivariate analysis compared to univariate analysis, we opted to extract the HRs and corresponding 95% CIs from multivariate analysis. Furthermore, the quality of the included studies was assessed using the Newcastle-Ottawa quality assessment scale (NOS). This evaluation serves to enhance the credibility and reliability of the findings presented in this academic paper. Data collection for each article were conducted independently by two authors (ZL and HZ). In the event of any disagreements, a third author (YZ) was consulted to resolve any discrepancies.

### 2.4 Statistical analysis

The results of the multivariate analysis, which include HRs and 95% CIs, were used to evaluate the prognostic effect of the CONUT score on the OS and RFS of patients with BTC. The Cochran’s Q test and I^2^ statistics were employed to evaluate the heterogeneity among studies. In cases where heterogeneity was significant (I^2^ > 50% and *P* < 0.10), a random effects model was utilized to combine the HRs and 95% CIs. If heterogeneity was insignificant, a fixed effects model was chosen. Subgroup analysis was performed to identify the sources of heterogeneity. Odds ratios (ORs) and corresponding 95% CIs were employed to determine the association between CONUT score and clinical characteristics. The statistical analyses were conducted using Stata software version 15.1 (Stata Corporation, College Station, TX, USA).

## 3 Results

### 3.1 Study characteristics

**Figure 1:**
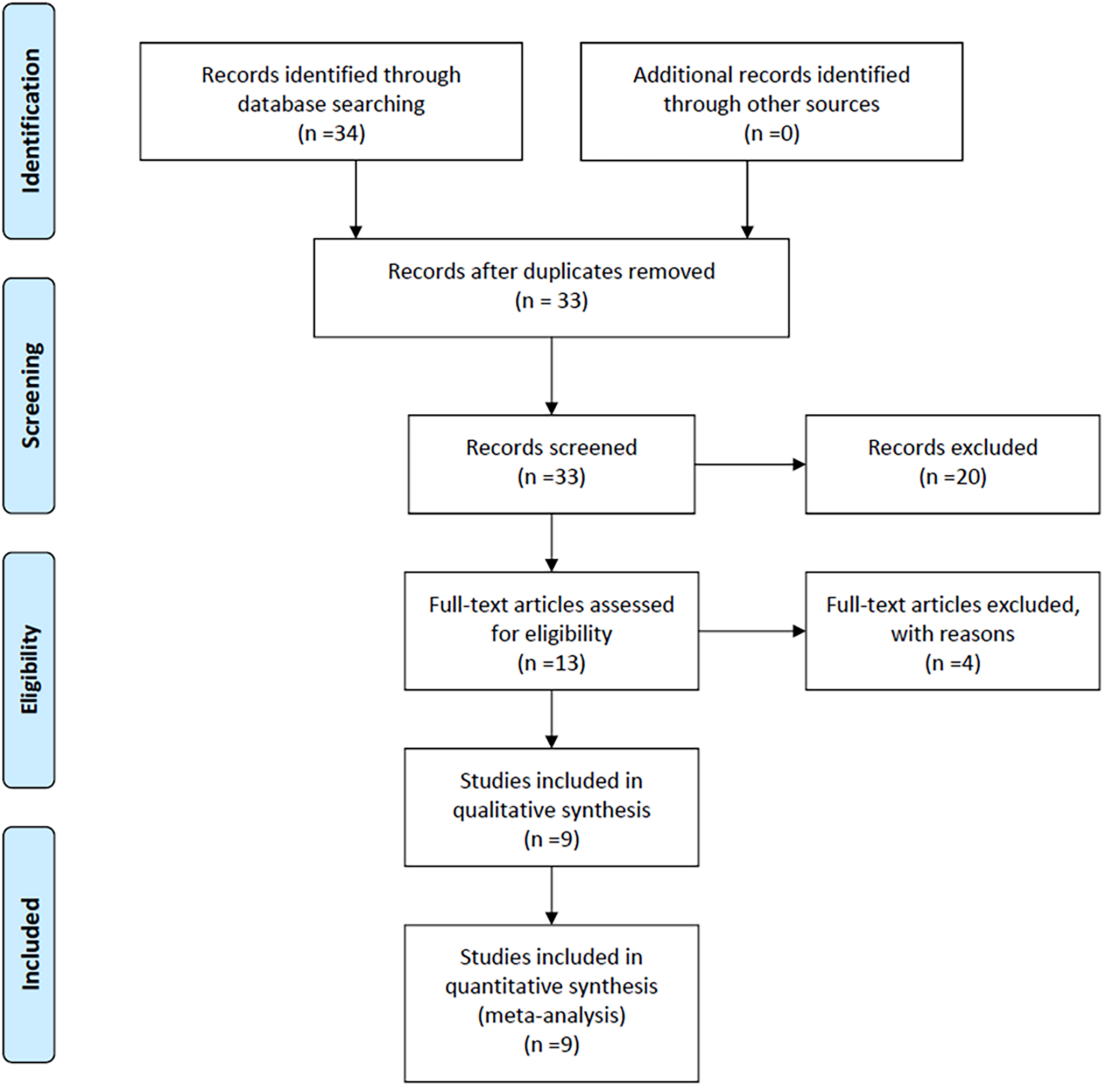
Flowchart of study selection for inclusion in the meta-analysis.

**Figure 1** illustrates that the initial literature search yielded 34 studies. After removing duplicate entries and excluding 20 studies that were not relevant to BTC and CONUT score, or comprised of posters, abstracts, or editorials, the titles and abstracts of the remaining studies were scrutinized. Subsequently, 13 studies were included for further screening. Among them, the studies by He (11) and Miyamoto (12) lacked survival outcome information related to the CONUT score. The study by Utsumi did not provide HRs data associated with the CONUT score (13). Additionally, Utsumi’s study included a subset of non-biliary cancer patients (14). Therefore, we excluded these 4 studies. Finally, the inclusion criteria were met by the remaining 9 studies (15–23).

The main clinical characteristics of the included studies are summarized in **Table 2**. The nine studies included 1409 patients from China and Japan who met the inclusion criteria. Five studies were conducted in Japan (17, 19–21, 23), and four studies were performed in China (15, 16, 18, 22). All of these studies were retrospective study. Except for patients in Cui et al. who received treatment by percutaneous transhepatic biliary stenting combined with ^125^I seed intracavitary irradiation (22), patients in other studies received surgery. Eight studies have reported the independent prognostic value of the CONUT score in predicting OS (16–23), and four studies have reported its independent prognostic value in predicting RFS (15, 16, 20, 21). The cut-off value of the CONUT score ranged from 2 to 4. The NOS scores ranged from 5 to 7.

**Table 2:** Characteristics of studies included in this meta-analysis.

Study, year	Country	Duration	Sample size	Tumor type	Study design	Treatment	Survival outcome	Cut-off	NOS
Asakura et al., 2022 (23)	Japan	2000-2019	169	ECC	Retrospective	Surgery	OS	3	6
Cui et al., 2018 (22)	China	2012-2017	73	HCCA	Retrospective	PTBS+ ^125^I	OS	2	5
Mito et al., 2023 (21)	Japan	2006-2020	224	BTC	Retrospective	Surgery	OS, RFS, DSS	4	6
Miyata et al., 2017 (20)	Japan	2002-2016	71	ICC	Retrospective	Surgery	OS, RFS	2	7
Shimizu et al., 2022 (19)	Japan	2002-2018	91	AVC	Retrospective	Surgery	OS	2	7
Sun et al., 2021 (18)	China	2002-2017	371	BTC	Retrospective	Surgery	OS	2	6
Terasaki et al., 2022 (17)	Japan	2002-2016	149	DCC	Retrospective	Surgery	OS	3	6
Wang et al., 2021 (16)	China	2010-2019	94	HCCA	Retrospective	Surgery	OS, RFS	3	7
Zheng et al., 2020 (15)	China	2012-2018	167	ICC	Retrospective	Surgery	OS, RFS	3	7

ECC, extrahepatic cholangiocarcinoma; OS, overall survival; HCCA, hilar cholangiocarcinoma; PTBS+ ^125^I, percutaneous transhepatic biliary stenting combined with ^125^I seed intracavitary irradiation; BTC, biliary tract cancer; RFS, relapse-free survival/recurrence-free survival; DSS, disease-specific survival; ICC, intrahepatic cholangiocarcinoma; AVC, ampulla of Vater cancer; DCC, distal cholangiocarcinoma.

### 3.2 CONUT score and OS

**Figure 2:**
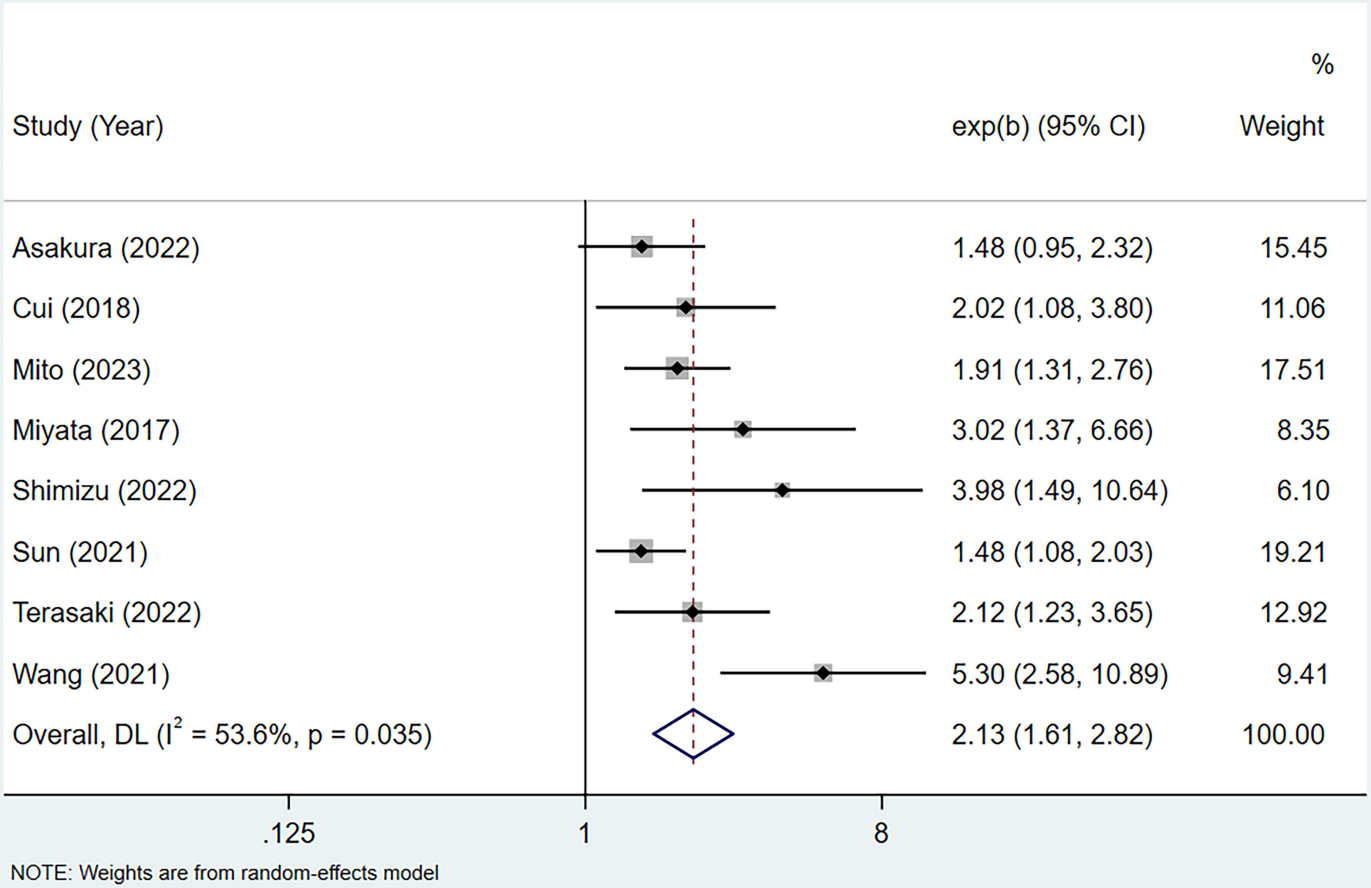
Forest plot of CONUT score in predicting OS in BTC.

8 studies (16–23), involving a total of 1242 patients diagnosed with BTC, reported the HRs and 95%CIs for OS. The study by Zheng et al. was excluded because only univariate analysis result was reported (15). The results of the heterogeneity test, characterized by an I² value of 53.6 and a *P*-value of 0.035, indicated significant differences among the studies included. Therefore, a random-effects model was employed. Based on a summary estimate of the HR, the high CONUT score was found to be a significant risk factor for patients with BTC (HR=2.13, 95% CI 1.61-2.82, *P*<0.0001) (**Figure 2**).

To account for the heterogeneity of OS, subgroup analyses were performed according to patients’ nationality, sample size, study quality, treatment method, tumor type, and CONUT score’s cut-off. As presented in **Table 3**, stratification by sample size revealed an HR of 1.67 (95% CI 1.37-2.03, *P* < 0.0001, I²=0%) for study with high sample size and an HR of 3.23 (95% CI 2.07-5.06, *P* < 0.0001, I²=27.3%) for study with low sample size. Stratification by study quality yielded an HR of 4.08 (95% CI 2.55-6.51, *P* < 0.0001, I²=0%) for high-quality studies and an HR of 1.70 (95% CI 1.40-2.05, *P* < 0.0001, I²=0%) for low-quality studies. These findings suggest that the sources of heterogeneity can be attributed to differential sample sizes and varying levels of study quality. Furthermore, significant correlations were observed between CONUT scores and OS within different subgroups.

**Table 3:** Subgroup analysis for OS.

Subgroup	No. of studies	HR(95%CI)	*P*	Heterogeneity I^2^(%)	Ph
Population					
China	3	2.38[1.17- 4.84]	0.017	80.4	0.006
Japan	5	2.00[1.54-2.61]	<0.0001	15.6	0.315
Sample size					
>140	4	1.67[1.37- 2.03]	<0.0001	0	0.558
≤140	4	3.23[2.07- 5.06]	<0.0001	27.3	0.248
Study quality					
High	3	4.08[2.55- 6.51]	<0.0001	0	0.587
Low	5	1.70[1.40- 2.05]	<0.0001	0	0.662
Treatment method					
Surgical	7	2.18[1.59- 2.98]	<0.0001	60.1	0.020
Palliative care	1	2.02[1.08- 3.80]	0.028	–	–
Cut-off					
2	4	2.11[1.37- 3.26]	0.001	48.1	0.123
3	3	2.43[1.24- 4.77]	0.010	77.0	0.013
4	1	1.91[1.31- 2.76]	0.001	–	–
Tumor type					
Mixed	2	1.65[1.29-2.10]	<0.0001	4.3	0.307
CCA	5	2.38[1.57-3.61]	<0.0001	57.6	0.051
AVC	1	3.98[1.49-10.64]	0.006	–	–

### 3.3 CONUT score and RFS

**Figure 3:**
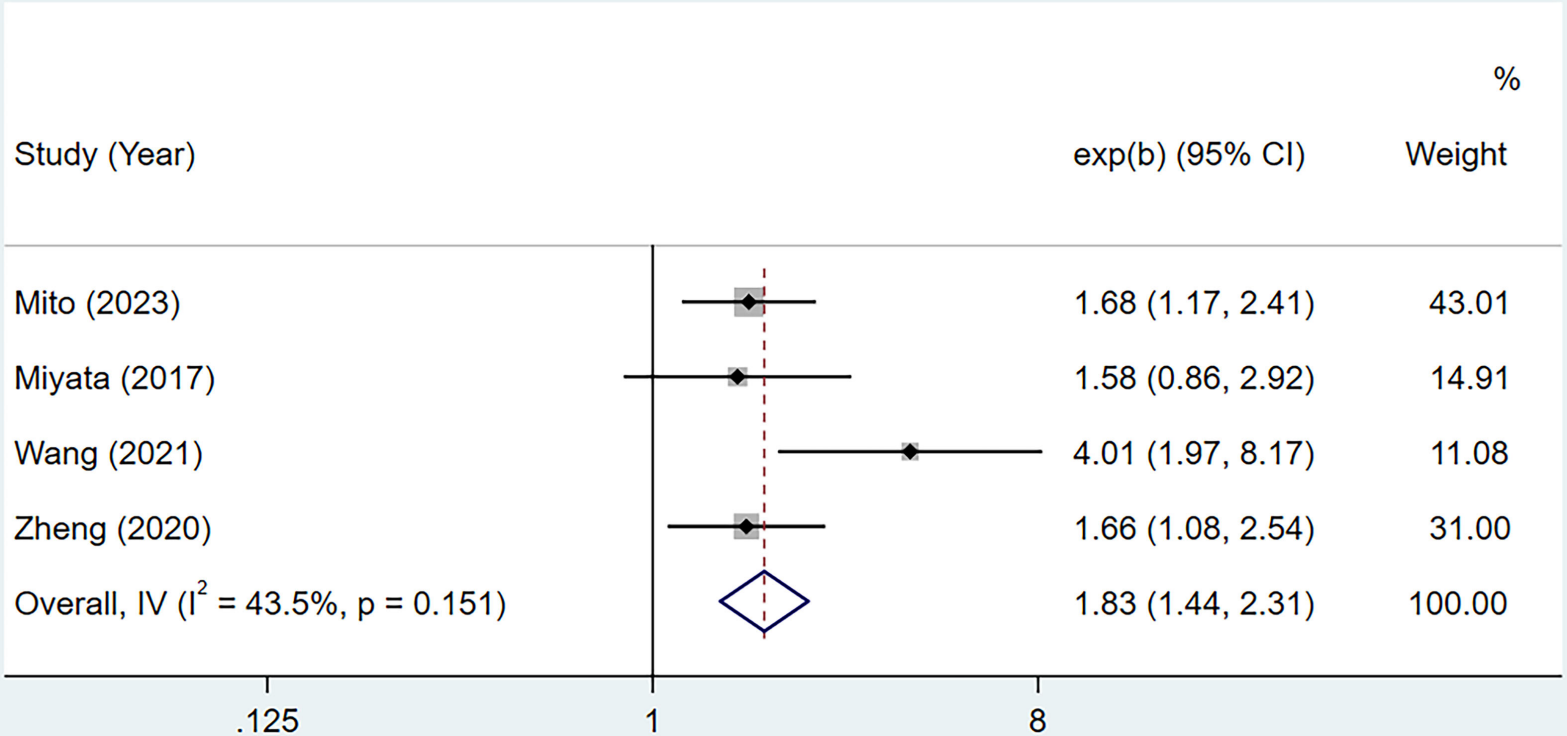
Forest plot of CONUT score in predicting RFS in BTC.

A total of 4 studies with 556 cases investigated the relationship between CONUT score and RFS in BTC (15, 16, 20, 21). Based on the analysis depicted in **Figure 3**, the pooled HR was found to be 1.83 (95% CI: 1.44-2.31, *P*<0.0001) without statistically significant heterogeneity (I^2 ^= 43.5%, *P*=0.151). These findings provide evidence that the CONUT score is significantly associated with RFS in BTC. After conducting a subgroup analysis, it was found that the relationship between a high CONUT score and poor RFS was not influenced by population, sample size, study quality, and tumor type. However, when using a cut-off value of 2 for the CONUT score, there was no significant correlation between a high CONUT score and poor RFS (HR:1.58, 95% CI: 0.86-2.92, *P*=0.144) (**Table 4**). We speculate that this lack of correlation may be due to the small number of studies included in this subgroup analysis.

**Table 4:** Subgroup analysis for RFS.

Subgroup	No. of studies	HR(95%CI)	*P*	Heterogeneity I^2^(%)	Ph
Population					
China	2	2.09[1.45-3.01]	<0.0001	77.0	0.037
Japan	2	1.65[1.21-2.26]	0.002	0.0	0.866
Sample size					
>140	2	1.67[1.27-2.20]	<0.0001	0.0	0.963
≤140	2	2.35[1.48-3.74]	<0.0001	73.5	0.052
Study quality					
High	3	1.94[1.42-2.66]	<0.0001	59.6	0.084
Low	1	1.68[1.17-2.41]	0.005	–	–
Cut-off					
2	1	1.58[0.86-2.92]	0.144	–	–
3	2	2.09[1.45-3.01]	<0.0001	77.0	0.037
4	1	1.68[1.17-2.41]	0.005	–	–
Tumor type					
Mixed	1	1.68[1.17-2.41]	0.005	–	–
CCA	3	1.94[1.42-2.66]	<0.0001	59.6	0.084

### 3.4 The association between CONUT and clinical characteristics

Based on data from 7 studies (15–21), this study investigated the association between CONUT score and various clinical characteristics. The results in **Table 5** indicate a significant association between CONUT score and jaundice (OR=1.60, 95% CI=1.14–2.25, *P*=0.006), poorly differentiated tumor (OR=1.43, 95% CI=1.03–1.99, *P*=0.03), pT3 and 4 stage of the tumor (OR=1.87, 95% CI=1.30–2.68, *P*=0.0007), and complications of Clavien-Dindo classification grade IIIa or higher (OR=1.79, 95% CI=1.03–3.12, *P*=0.04).

**Table 5:** The association between CONUT score and clinicopathological features in patients with BTC.

Factors	Studies(n)	OR(95%CI)	*P*	Heterogeneity I^2^(%)	Ph	Effects model
jaundice(yes vs no)	2	1.60[1.14-2.25]	0.006	0	0.43	Fixed
degree of differentiation(poorly vs medium-high)	3	1.43[1.03-1.99]	0.03	0	0.38	Fixed
pT stage (T3,4 vs T1,2)	4	1.87[1.30-2.68]	0.0007	0	0.70	Fixed
complications (CD≥IIIa)(yes vs no)	3	1.79[1.03-3.12]	0.04	34	0.22	Fixed

CD, Clavien-Dindo classification.

### 3.5 Sensitivity analysis

**Figure 4:**
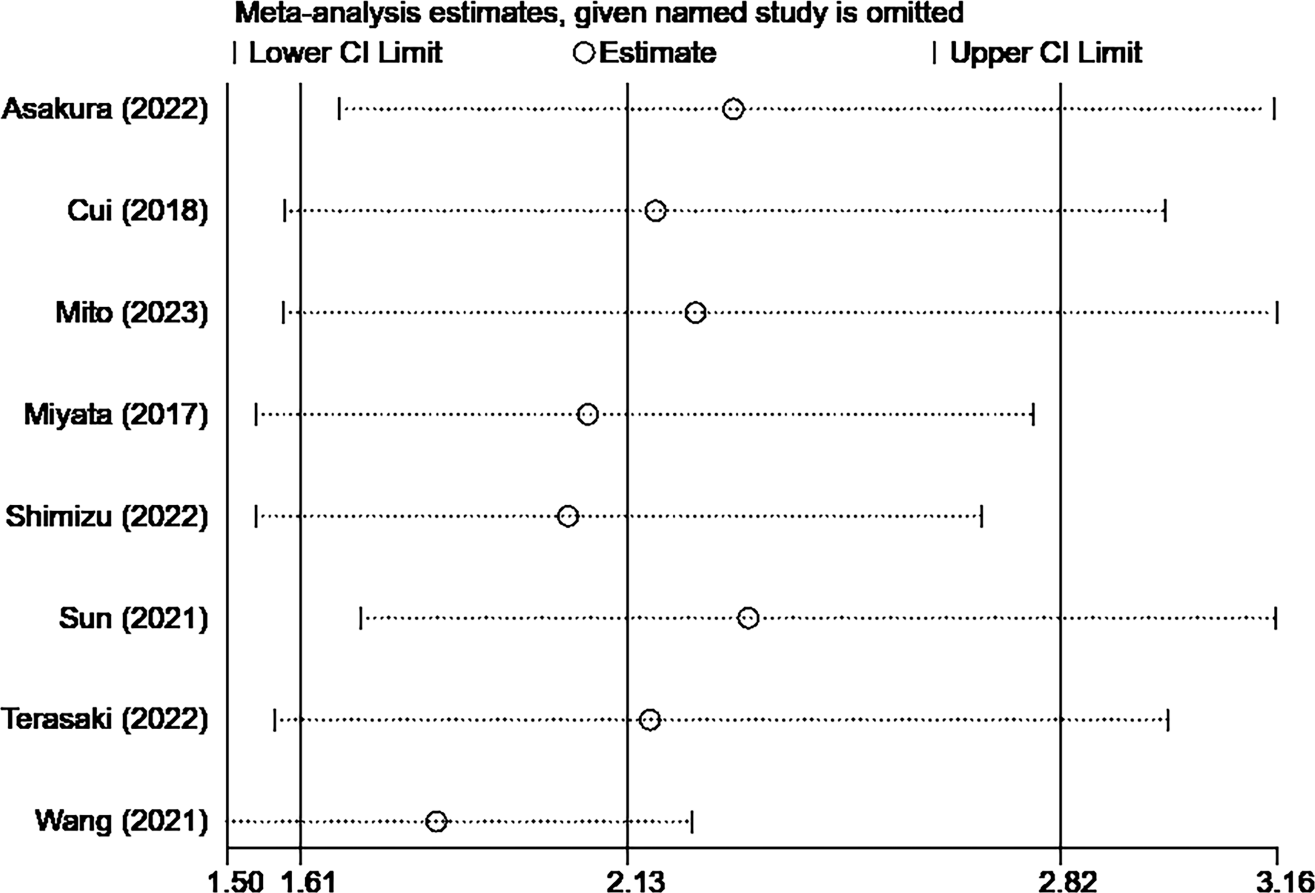
Sensitivity analysis result of OS.

**Figure 5:**
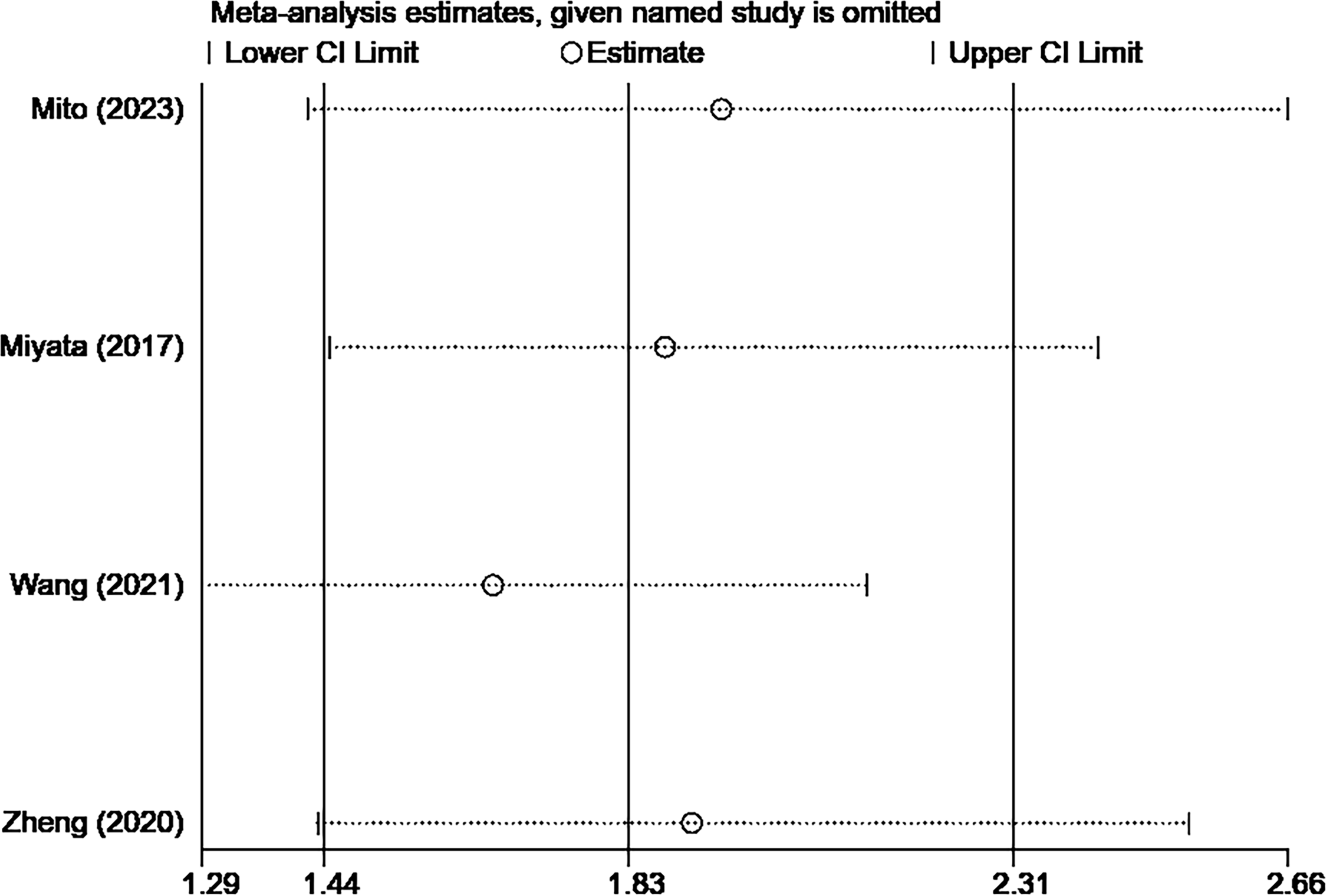
Sensitivity analysis result of RFS.

The sensitivity analysis showed that the overall results remained stable and reliable even when one study was omitted, indicating no significant changes (**Figures 4**, **5**).

## 4 Discussion

In recent years, researchers have conducted extensive investigations into the potential prognostic impact of inflammatory and nutritional markers in cancer patients. In this study, our objective was to examine the prognostic value of the CONUT score in patients diagnosed with BTC. However, given the inconsistent and contradictory findings from the existing studies, we employed the meta-analysis approach to elucidate the potential role of the CONUT score in predicting prognosis in BTC patients.

Previous meta-analyses, which were limited in terms of literature coverage and solely focused on cholangiocarcinoma, have omitted to elucidate the association between the CONUT score and BTC outcomes in both short and long terms, thereby casting doubt on the credibility of their findings. In a meta-analysis by Takagi and colleagues (24), no correlation was observed between the CONUT score and postoperative complications in cases of hepatobiliary pancreatic malignancies. Our study reveals a significant correlation between a high CONUT score and postoperative complications of BTC (OR = 1.79, 95% CI: 1.03-3.12, *P* = 0.04). We further demonstrate that the CONUT score serves as an independent prognostic factor for OS and RFS in patients with BTC. Specifically, our findings suggest that the CONUT score may be associated with preoperative jaundice, postoperative tumor differentiation, pT stage, and complications in these patients. Moreover, through a sensitivity analysis, we obtained stable and reliable meta-analytical results. Our study represents the first to systematically investigate the prognostic significance of the CONUT score in BTC.

The CONUT score, which encompasses serum albumin, cholesterol, and peripheral blood lymphocyte counts, is employed as a prognostic tool for patients with BTC. However, the underlying mechanism through which it influences patient prognosis remains inadequately understood. A wealth of pertinent literature suggests that tumor development is inextricably linked to both immune function and nutritional status (25, 26), Research findings suggest that decreased levels of albumin are linked to unfavorable outcomes in malignant conditions (27, 28), decreased levels of albumin may impair the immune function of the body, leading to a reduction in the immune response against cancer cells and promoting tumor development (29, 30). Cholesterol is involved in the fundamental construction of cell membranes and maintains cellular physiological functions through intracellular signal transduction. When cholesterol levels decrease, it indicates insufficient energy storage and metabolic imbalance (31). The activity of low-density lipoprotein receptors is reported to be elevated in cholangiocarcinoma cells, indicating that hypocholesteremia may result from excessive uptake of cholesterol by tumor cells (32). Lymphocytes, as a crucial immune component, have been shown to inhibit the proliferation, migration, and invasion of cancer cells. Consequently, a higher CONUT score is associated with decreased patient survival (33).

Several meta-analyses have previously investigated the prognostic value of the CONUT score in malignant tumors. Kosei et al. conducted a meta-analysis that included 2601 patients, revealing a significant association between high CONUT score and poor prognosis in patients with colorectal cancer (10). A meta-analysis involving eight studies has demonstrated the correlation between high CONUT score and OS, cancer-specific survival, and RFS in patients with urothelial carcinoma who have undergone systematic treatment (34). A meta-analysis conducted by Zhang et al. showed that the preoperative CONUT score can serve as an independent prognostic factor for long-term survival in patients with gastrointestinal tumors, which can assist in predicting their postoperative survival status (35). Our meta-analysis demonstrates that the CONUT score has consistent prognostic value with other cancer types in terms of OS and RFS in patients with BTC. Additionally, we found a significant correlation between higher CONUT score and clinical and pathological characteristics of BTC. Based on these findings, the CONUT score can provide important evaluation for the treatment of patients with BTC, helping clinicians to develop more personalized treatment plans. Therefore, caution should be taken in the treatment strategy for BTC patients with high CONUT score.

This meta-analysis has several limitations. Firstly, all included studies were retrospective and the relatively small sample size necessitates further improvement of the quality of evidence. Secondly, the study only included patients from Asia, which may introduce region-based biases. Thirdly, there was inconsistency in the critical values of the CONUT score used in different studies. Finally, more multi-center, large-scale, prospective studies are required to validate these findings due to limited research reports on disease-specific survival and related areas.

## 5 Conclusion

The CONUT score is a reliable, simple, easily obtainable, and cost-effective index for predicting the prognosis of patients with BTC. It is an independent prognostic factor for OS and RFS in this patient population and has correlations with preoperative jaundice, postoperative tumor differentiation, pT stage, and complications. The European Society for Clinical Nutrition and Metabolism suggests employing a range of tools, including nutritional risk screening tool 2002, for the periodic evaluation of nutritional status and repeat assessment during cancer diagnosis (36). However, the use of current nutritional screening tools has not reached a consensus. The CONUT score can complement the selection of nutrition screening tools. Preoperative use of the CONUT score for nutritional risk stratification and individualized treatment based on different nutritional statuses can potentially improve treatment outcomes, particularly in individuals who are elderly and feeble, who may be at greater risk, and who have certain chronic diseases. A significant inference might also be generated by current research trends. The emergence of novel biomarkers that have caught the attention of researchers and use of these biomarkers to determine different treatment options for patients holds promise as a potential future therapeutic target. Although there is a lack of research on how improvements in CONUT score values relate to differences in disease prognosis, it is undeniable that the CONUT score reflects nutritional deficiency and inflammatory responses in the body. As a potential tool for nutritional risk stratification, further research is still needed for validation.

## Author contributions

ZL and JL designed the study. ZL, HZ, and YZ established the process of literature selection and screened the abstracts and articles. MY, YC and HZ analyzed data and wrote the main manuscript. All authors contributed to the article and approved the submitted version.
